# Microplastics and nano-plastics as emerging gut–brain axis disruptors: mechanistic insights, health implications, and future direction

**DOI:** 10.3389/fpubh.2026.1885585

**Published:** 2026-08-07

**Authors:** Swagat Suman Patra, Sunita Panda, Jyotirmayee Turuk, Sanghamitra Pati

**Affiliations:** ICMR-National Institute of Health Research, Bhubaneswar, India

**Keywords:** blood-brain barrier, dysbiosis, gut–brain axis, microplastic, nanoplastics, neuroinflammation

## Abstract

Plastics are synthetic polymers widely used for their versatility, longer shelf life, and low cost. However, the non-biodegradable nature of conventional plastics has led to accumulation of microplastics (MPs; <5mm) and nanoplastics (NPs; <100 nm) in aquatic, terrestrial, and atmospheric environments. MNPs are composed of polyethene, polypropylene, polystyrene, polyvinyl chloride, and polyethene terephthalate. Human exposure to micro and nanoplastics occurs mainly through ingestion, inhalation, and rarely through dermal contact. The gastrointestinal tract is the potential accumulation site for such particles, which leads to microbial dysbiosis, disturbs intestinal barrier integrity and induces oxidative stress and inflammation. These disruptions lead to diseases related to the gastrointestinal tract, chronic systemic inflammation and affect the gut-brain axis with neuroinflammatory consequences. This review highlights gut dysbiosis and intestinal barrier disruption as a crucial gateway mediating the systemic effects of MNPs. On exposure to MNPs, gut microbial composition is altered by reducing beneficial short-chain fatty acid-producing bacteria such as *Faecalibacterium, Roseburia*, and *Bifidobacterium*, while stimulating the growth of opportunistic pathogens such as Desulfovibrio spp. Subsequent reductions in short-chain fatty acid generation induce lipopolysaccharide release and downregulation of tight junction proteins that compromise intestinal barrier integrity and expedite the translocation of MNPs, microbial products, and pro-inflammatory determinants into systemic circulation. MNPs then cross the blood-brain barrier by receptor-mediated routes, paracellular diffusion, or transcytosis, which is exacerbated by inflammation and oxidative stress. Excessive ROS further cause mitochondrial dysfunction, neuroinflammation, microglial activation, neurotransmitter imbalance, and pathological protein aggregation. This is a narrative review, focusing on integrating current evidence on the role of MNPs in disturbing the gut-brain axis through gut dysbiosis, intestinal barrier dysfunction, increased oxidative stress, inflammation, and altered neurochemical signaling. It further highlights the consequences of these changes in systemic health, identifies key knowledge gaps, and outlines future research priorities to better understand and mitigate the health risks.

## Graphical Abstract

**Figure:**
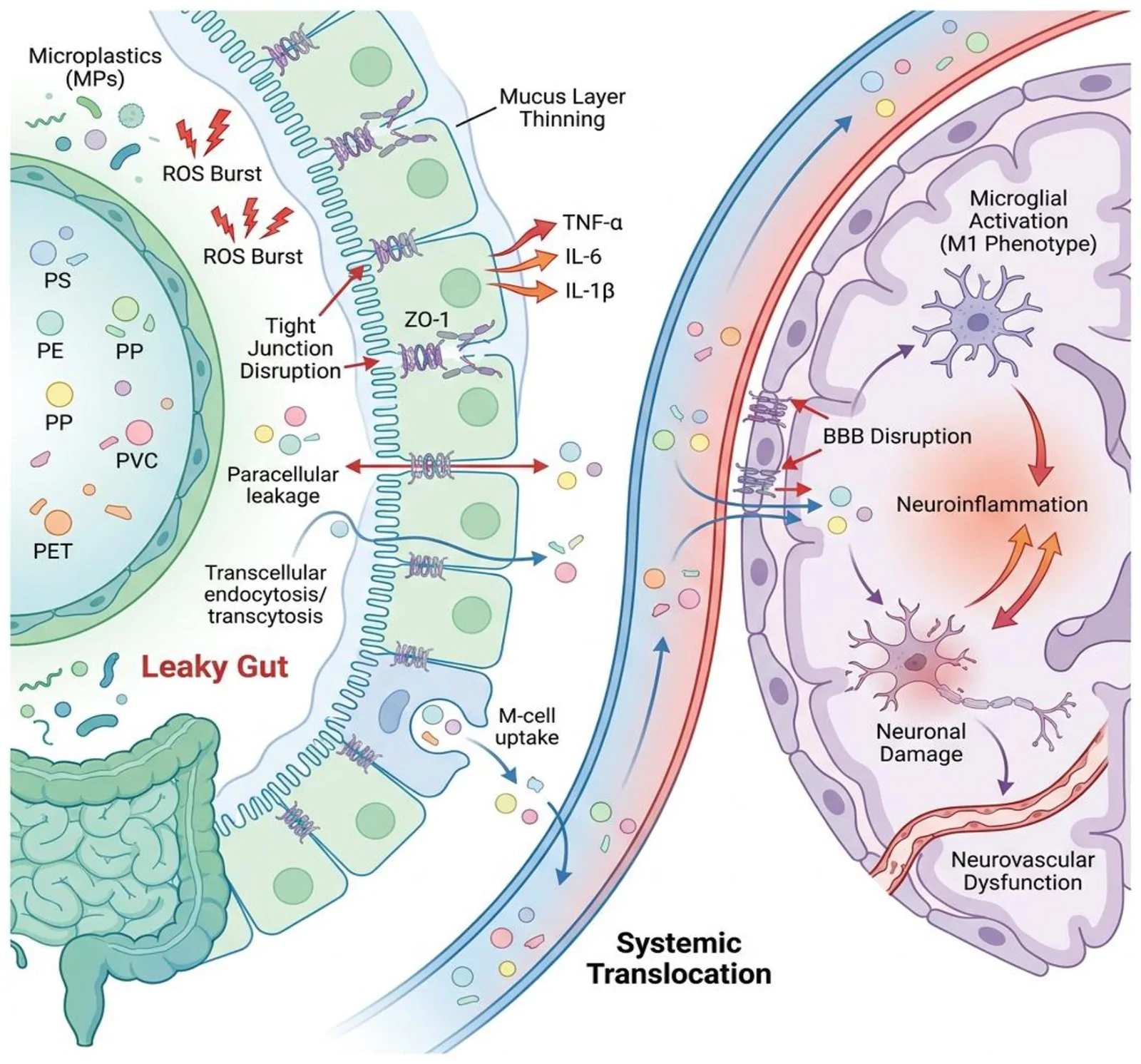
Figure illustrating proposed mechanism of gut-brain axis disruption by MNPs. Following exposure to MNPs that accumulate in GI tract and induce oxidative stress and dysbiosis, disruption of epithelial tight junctions. This changes, resulting in leaky gut and increased permeability, lead to translocation of MNPs, microbial metabolites, cytokines into systemic circulation and disruption of GBA. BBB penetration of MNPs into the central nervous system promotes microglial activation, neuroinflammation, neuronal injury, and neurovascular dysfunction.

## 1 Introduction

Microplastics (MPs) and nanoplastics (NPs), composed of plastic fragments smaller than 5 mm and 1 μm, respectively, are a subtle and pervasive type of environmental pollutant that originates from the breakdown of larger plastics, manufacturing processes, synthetic textiles, and everyday products such as tire and cosmetics (1–5). MPs and NPs have affected ecosystems all over the world since they were first discovered in marine habitats more than 20 years ago (3, 6, 7). These are found ubiquitously in the environment, from air, soil, and water bodies, to even isolated polar areas (3, 4, 8). This challenge is further intensified by the production of over 400 million tons of plastic worldwide each year. Without stringent waste management strategies, plastic emissions into the environment are projected to rise to 10–40 million tons annually by 2040, driving a dramatic surge in the accumulation of microplastics and nanoplastics across the environment and amplifying their ecological and public health consequences (3, 4). These particles not only endure because of their chemical stability, but they also serve as carriers of hazardous additives (like phthalates and bisphenols) and adsorbed ambient contaminants (such as heavy metals and persistent organic pollutants), increasing the dangers to the environment and human health (1, 3, 4, 8, 9).

Humans are primarily exposed to microplastics (MPs) and nanoplastics (NPs) through ingestion of contaminated food and drinking water, by inhaling airborne particles, and direct contact with skin. Recent findings of such contaminants in human tissues, such as the brain and placenta, MNPs concentrations have been found in the brain tissue of dementia patients compared to age-matched neurologically healthy controls, with burdens 2.4–7.5 times higher in affected individuals and preferential accumulation in the hippocampus and frontal cortex. These findings support a plausible dose-dependent association with neurological impairment (4, 10, 11). The gut-brain axis (GBA), a bidirectional communication network including neuronal, endocrine, and immunological pathways that integrates digestive balance with central nervous system (CNS) function, is disrupted by MPs and NPs, according to emerging research (4, 10, 12, 13). Consumed MPs change the makeup of the gut microbiota, causing dysbiosis marked by decreased synthesis of short-chain fatty acids and an increase in pathogenic taxa (such as *Desulfovibrio spp*.), which increases systemic inflammation and intestinal barrier permeability (3, 4, 8, 14). This “leaky gut” facilitates MP translocation into circulation, enabling blood-brain barrier (BBB) crossing via endocytosis, paracellular, or Trojan horse pathways, where they accumulate in neural tissues and trigger neurotoxicity (2, 13, 15, 16).

Reactive oxygen species (ROS) production in excess, mitochondrial dysfunction, and neuroinflammation through stimulation of microglial cells and cytokine release (e.g., IL-1β, TNF-α) are the mechanisms by which MP/NP exposure causes oxidative stress (3, 4, 8, 16–18). The hallmarks of neurodegenerative diseases like Alzheimer's disease (AD), Parkinson's disease (PD), and amyotrophic lateral sclerosis (ALS) include synaptic impairment, neurotransmitter dysregulation (e.g., decreased serotonin and dopamine), and accumulation of proteins, including amyloid-β (Aβ) and α-synuclein (4, 17–19). Exacerbated neuronal death after ischemia, cognitive abnormalities, and motor impairments after MP accumulation are shown in animal models, with offspring showing transgenerational consequences (2, 3, 5, 20). Human epidemiological data, though limited, correlate higher MP burdens with dementia risk, potentially via GBA-mediated pathways like altered vagus nerve signaling and lipopolysaccharide translocation (4, 17).

Significant gaps still exist despite these advancements: mechanistic studies frequently ignore co-exposures with contaminants, real-world particle heterogeneity (such as size, shape, and polymer type), and sensitive groups such as children and older adult(s) people (5, 15, 16). Furthermore, although membrane filtration and bioremediation are promising water treatment technologies for MP removal, their capacity for expansion and effectiveness against NPs are still little understood (3). This review synthesizes current evidence on MP/NP interactions with the GBA and CNS, elucidating exposure routes, toxicological mechanisms, and disease associations. By addressing these gaps, we aim to inform risk assessments, policy interventions, and future research directions to mitigate this emerging threat to neurological health. This review primarily focuses on gut dysbiosis and its impact on brain communication, as well as bioaccumulation of MPs in brain tissue. It comprehensively integrates current evidence on GBA disruption due to MNP exposure. It interlinks environmental exposure, gut accumulation and dysbiosis, compromised gut integrity and systemic translocation, blood–brain barrier impairment, and neurodegenerative mechanisms into an integrated mechanistic frame while critically identifying knowledge gaps and future research scopes.

## 2 Sources, types, bioaccumulation and routes for human exposure

Over 400 million tons of plastic are produced worldwide each year, and both deliberate manufacture and environmental deterioration result in a range of micro- and nanoscale pieces (3, 21). MPs and NPs are widely distributed across ecosystems as a result of the intricate interaction between natural processes and human activity. These particles experience physical, chemical, and biological changes after being released, which affect their bioavailability, transit, and permanence. Ingestion, inhalation, and skin contact are the main ways that humans are exposed, and bioaccumulation increases the dangers at all trophic levels. This section explains the origins of MPs and NPs, the dynamics of their surrounding environment, and how they enter human systems to estimate exposure dosages using available data.

### 2.1 Primary vs. secondary MNPS

Based on where they came from, MPs and NPs are divided into both primary and secondary groups. For industrial uses, such as microbeads in beauty products, exfoliants in products for personal use (including toothpaste and face washes), and particles in cleaning solutions, primary MNPs are purposefully produced at micro- to nanoscale sizes (11, 17, 21, 22). For example, estimates indicate that up to 94,500 pieces per face scrub application infiltrate wastewater systems, making microbeads from cosmetics a major source of aquatic pollution (22). About 35% of marine MPs come from synthetic fabrics, including as clothes and furniture, which release primary microfibers after washing (22, 23). Another significant primary source is tire wear particles, which are produced by road abrasion and account for 28%−42% of MPs in urban runoff (22, 24). Primary NPs are also released into the environment by industrial operations, including 3D printing and the manufacture of plastic pellets (nurdles) (22).

Secondary MNPs, on the other hand, are created when bigger plastic objects break apart due to weathering. Packaging, bottles, and fishing gear are examples of macroplastics that are broken down into tiny pieces by processes such UV photodegradation, oxidation by heat, mechanical abrasion, and biodegradation (5, 17, 22, 23, 25). For instance, in soil and marine habitats, respectively, abandoned nets used for fishing and agricultural mulching films break down into secondary MPs (22–24). Fragmentation rates are influenced by polymer types: PS and PE break down more quickly under UV light than PVC or PET (22, 26). By accelerating chemical breakdown, co-exposure to pollutants like metals or organic pollutants can hasten secondary formation (13, 21, 22). Collectively, understanding the origins of MNPs is important to tailor effective strategies to control their human exposure and associated health risk.

### 2.2 Global production and waste projections

MPs and NPs are proliferating due to the exponential increase in plastic manufacturing. Global output increased by 4% from 2021 to 400.3 million tons in 2022, and under business-as-usual scenarios, it is predicted to reach 1,200 million tons by 2060 (2, 3, 22). Production is dominated by Asia (51%), North America (19%), and Europe (16%), with packaging (44%) and textiles (14%) being the top industries (22, 27). This increase is reflected in the amount of waste generated: 353 million tons were created in 2019, of which only 9% were recycled, 19% were burned, and 50% were landfilled; the remaining 22% were mishandled and leaked into the environment (22, 27). Unmanaged trash is expected to increase MP/NP buildup to 10–40 million tons per year by 2040 (3, 4, 22, 27).

Every year, 8–14 million tons of plastic garbage are dumped into marine habitats, mostly from land-based sources such as littering and poor waste management (22, 23). Eighty percent of oceanic MPs come from terrestrial inputs, such as urban runoff and agricultural plastics (22, 23). According to projections, the amount of plastic debris in the ocean may triple by 2040 and reach 29 million tons per year if no action is taken (22, 27). These patterns demonstrate how urgently circular economy approaches are needed to reduce the creation of primary and secondary MP/NP (22, 27). Therefore, adoption of sustainable production and effective waste management practices is the need of the hour to reduce the global burden of MNPs that pose challenges for human health.

### 2.3 Routes of human exposure and dosimetry

**Figure 1:**
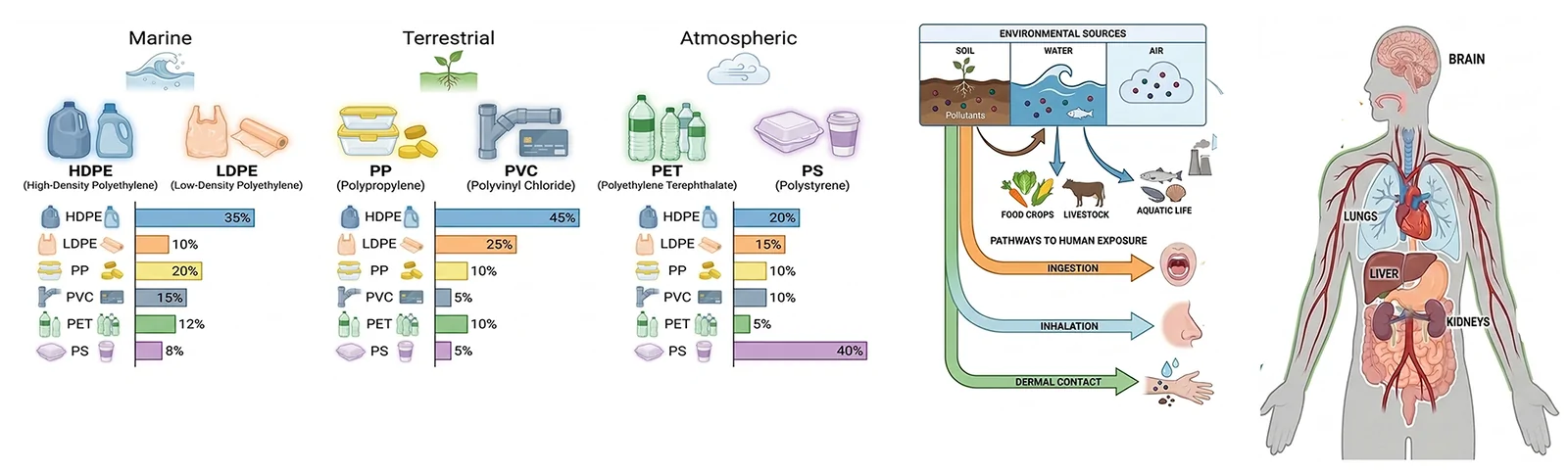
**Schematic representation of various types of plastic wastes as MNPs and their distribution across the ecosystem, bioaccumulation and route of exposure to the human system**. Major polymer wastes such as high-density polyethylene (HDPE), low-density polyethylene (LDPE), polypropylene (PP), polyvinyl chloride (PVC), polyethylene terephthalate (PET), and polystyrene (PS), are widely distributed across the environment. Exposure to human systems occurs through ingestion of contaminated food and water, inhalation of airborne wastes, and by dermal contact. MNPs, can cross biological barriers and enter through systemic circulation, and accumulate in various organs to exert its toxicity. The figure has been incorporated in the manuscript is generated with the help of an AI tool from figurelabs.ai.

Humans come into contact with MNPs through a variety of means, the most common being ingestion, which contributes 39,000–52,000 particles per year through tainted food (such as seafood, salt, and bottled water) and drinks (17, 21, 28–30). In high-consumption groups, seafood alone contributes 11,000 particles annually, whereas bottled water adds 4,000–90,000 particles annually (17, 21) (Figure 1). MPs from processing and packaging deterioration are present in beverages, such as milk, beer, and soft drinks, in quantities of up to 40 particles/L (21, 30). Indoor inhalation exposes people to 21–26 particles per day, mostly from dust and synthetic fabrics; urban residents can inhale up to 272 MPs/m^3^ yearly (28, 29, 31). NPs (<100 nm) penetrate skin barriers through clothes and cosmetics, resulting in dermal contact, albeit less quantifiable (21, 25, 28).

Adults consume 0.1–5 g of MPs each week through mixed pathways, which is equal to the mass of a credit card, according to varying dosimetry estimations (21, 28, 29). Due to their lower body weight and hand-to-mouth habits, children are more exposed than adults (28). Fecal MPs can reach 1,000 particles/g in occupational groups like textile workers (28). These estimations highlight the necessity of uniform measurement techniques to improve exposure evaluations (17, 21, 28, 31).

### 2.4 Bioaccumulation and trophic transfer

MPs and NPs increase human exposure by bioaccumulating in organisms and transferring across food webs. Fish and shellfish in aquatic systems get NPs (0.001–0.1 μm) from plankton, with biomagnification factors of up to 10 times each trophic level (2, 4, 15, 17, 31, 32). Earthworms and other soil creatures absorb MPs and transport them to crops, a process known as terrestrial bioaccumulation. MP concentrations of 0.6–10 particles/g are found in human tissues, such as the placenta, breast milk, and blood, suggesting transgenerational transmission (4, 15, 17, 29). Higher loads in dementia patients are revealed by autopsy investigations, indicating CNS buildup (15, 17). Phthalates and other co-adsorbed pollutants increase toxicity during trophic transmission (1, 4, 17, 18, 20). Taken together, bioaccumulation and biomagnification facilitate the transfer of MNPs, and it needs evaluation of long-term health risk.

### 2.5 Common MNPs' physicochemical characteristics and how they affect transmembrane transfer

Intestinal absorption, barrier translocation, microbiome interactions, and neurotoxicity are all significantly influenced by the physicochemical characteristics of micro- and nanoplastics (MNPs), including polymer type, size, shape, surface charge, hydrophobicity, and crystallinity (1, 18). Polyethene (PE), polypropylene (PP), polystyrene (PS), polyvinyl chloride (PVC), and polyethene terephthalate (PET) are the most common polymers (22) (Figure 1).

Due to their high hydrophobicity and flexibility, PE particles exhibit increased systemic translocation of smaller PE-NPs (less than 500 nm) through leaky gut, which increases brain accumulation and oxidative stress (3, 15). Semicrystalline PP fragments and fibers create protein coronas that promote M-cell uptake and paracellular transport, aggravating BBB crossing and gut dysbiosis (18, 22).

Positively charged PS-NPs (<200 nm) show 2–5 × greater endocytosis in enterocytes and transcytosis across the BBB, triggering microglial activation and protein aggregation. PS (typically spherical) displays size- and charge-dependent uptake (1, 33). PVC particles, which are high in plasticisers (such DEHP), increase paracellular leakage and neuroinflammation by inhibiting PPAR-γ, which downregulates tight junctions (ZO-1, occludin) (3). Weathered particles increase contaminant adsorption and CNS entrance, whereas moderately hydrophilic PET fragments cause mucus layer disruption by size-dependent translocation (2).

In general, transmembrane transfer efficiency across intestinal and blood-brain barriers is increased by smaller size (<200 nm), positive surface charge, irregular forms, and hydrophobicity, exacerbating disturbance of the gut-brain axis (18, 22).

## 3 Translocation from gut to brain: barrier crossing mechanism

**Figure 2:**
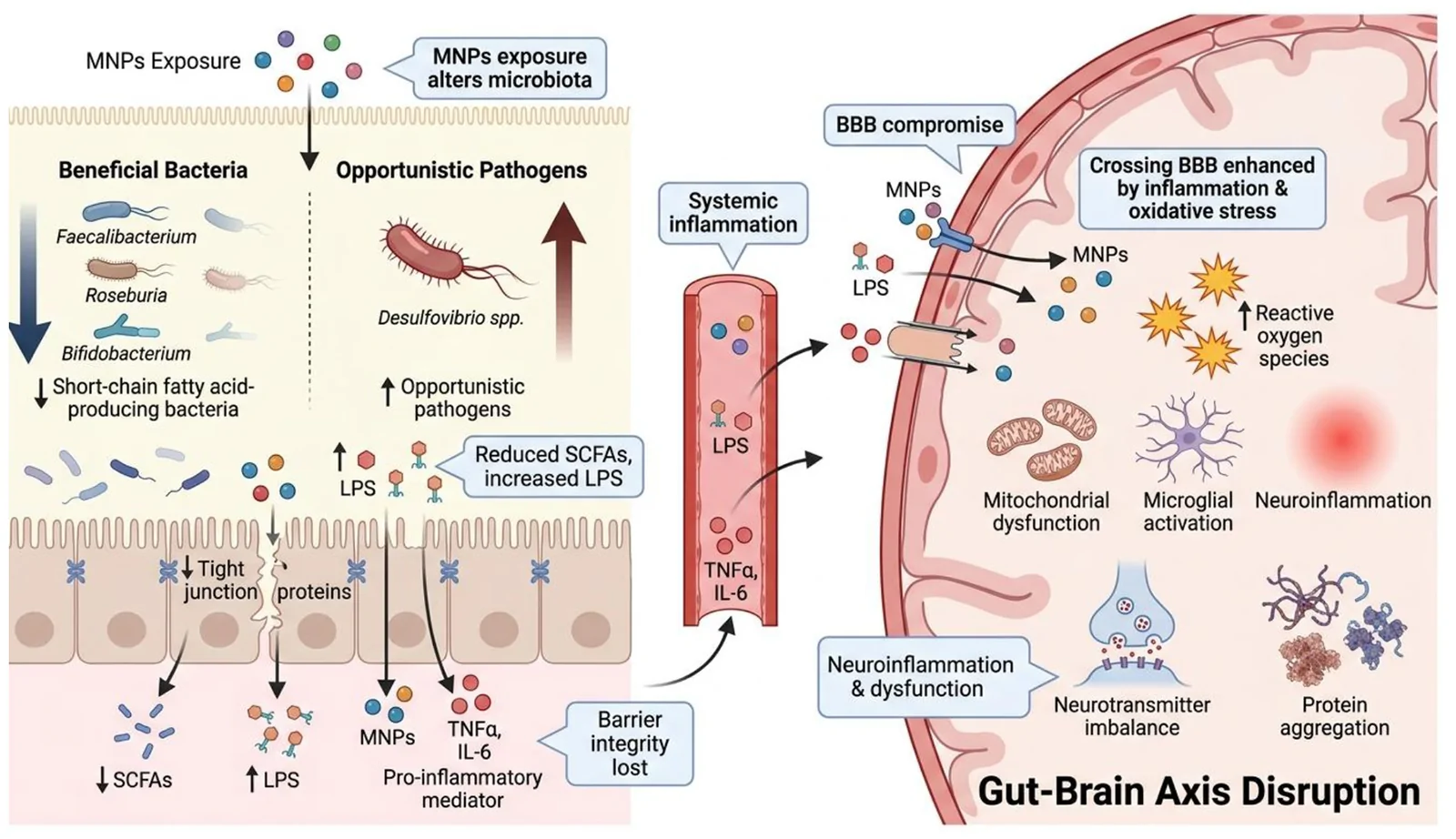
**Schematic representation of the translocation of MNPs from compromised gut to brain**. MPs/NPs induce gut dysbiosis and intestinal barrier disruption allowing MNPs, pathogens, and inflammatory mediators to enter systemic circulation. Through endocytosis, paracellular transport, and Trojan horse processes facilitated by immune cells, they move across the blood-brain barrier (BBB). Another factor is direct messaging via the gut-brain axis, or vagus nerve. Accumulated MPs/NPs in the brain cause neuroinflammation by activating microglia and releasing ROS, TNF-α, and IL-1β. (The figure incorporated in the manuscript is generated with the help of an AI tool from figurelabs.ai).

After consumption, microplastics (MPs) and nano-plastics (NPs) go from the gastrointestinal lumen into the bloodstream and cross the blood-brain barrier (BBB), allowing them to build up in brain regions (2, 4, 14, 15, 17, 18) (Figure 2). This multi-step translocation process exploits compromised intestinal integrity, immune-mediated transport, and size-dependent physicochemical properties of plastic particles. Emerging evidence elucidates three primary mechanisms endocytosis, paracellular leakage, and Trojan horse phagocytosis each modulated by particle size, surface chemistry, and co-exposure to gut dysbiosis or adsorbed pollutants (2, 15, 16).

### 3.1 Endocytosis in enterocytes and M cells

NPs (<200 nm), due to their nanoscale dimensions, are more frequently internalized via clathrin- or caveolae-mediated endocytosis in enterocytes and M cells of Peyer's patches (2, 15, 16, 33). Translocation efficiencies of 10%−30% across Caco-2/HT29-MTX co-culture monolayers have been demonstrated for polystyrene NPs (50–100 nm), which are substantially greater than MPs (>1 μm) (33, 34). Surface charge is important: because of electrostatic interactions with the negatively charged glycocalyx, positively charged amine-modified NPs show 2–5 times greater absorption than neutral or carboxylated counterparts (16, 33). After internalization, 40%−60% of the time, NPs avoid lysosomal breakdown by being transported by early endosomes and discharged into the lamina propria through exocytosis (15, 34). Long-term exposure increases translocation rates by further upregulating endocytic pathways through ROS-mediated activation of PI3K/Akt signaling (16). The nanoscale size of NPs facilitates the breaching of intestinal barriers, entering the systemic circulation and reaching the distal organs and thus making them more hazardous than microplastics.

### 3.2 Paracellular leakage via tight junction disruption

Tight junction integrity is compromised by MP/NP-induced gut dysbiosis and inflammation, allowing MPs and NPs to translocate paracellularly (4, 18, 19, 35). Zonula occludens-1 (ZO-1) and occludin expressions are downregulated by 40%−70% in murine models when *Desulfovibrio spp*. overgrowth, which is frequently seen after MP exposure, decreases butyrate-producing taxa (such as *Faecalibacterium*) (4, 9). MPs up to 10 μm can move between enterocytes thanks to this “leaky gut” phenotype, which increases intestinal permeability (as determined by FITC-dextran flow) three to five times (35). By inhibiting PPAR-γ, co-exposure to phthalates adsorbed on MPs increases this impact and further destabilizes junctional complexes (18).

### 3.3 BBB penetration pathways

MPs and NPs use a variety of methods, including transcytosis, paracellular diffusion, and receptor-mediated transport, to cross the blood-brain barrier once they reach the cerebral endothelium (11, 15, 16). According to Dihydroethidium (DHE) fluorescence and low-density lipoprotein receptor (LDLR) mRNA expression, PEGylated polystyrene nanoparticles (100 nm) exhibit 3.1% brain absorption in zebrafish through Low-Density Lipoprotein (LDL) receptor-mediated transcytosis, a mechanism that is 2.8-fold increased during MP-induced oxidative stress (15). Bigger MPs (1–5 μm) depend on inflammation-induced paracellular leakage. After 24 h of exposure to 2 μm PS-MPs (10 μg/mL) with TNF-α co-stimulation, MP-triggered cytokine storms reduce claudin-5 by 58% and occludin by 42% in human BBB endothelial monolayers (hCMEC/D3). This increases Transepithelial electrical resistance (TEER) reduction by 310% and FITC-dextran permeability by 340% (16). Penetration is further modulated by adsorbed environmental contaminants: By downregulating metallothionein-2 and activating Myosin light chain kinase (MLCK), lead (Pb)-coated MPs compromise BBB integrity, allowing 3 μm particle penetration in rat models (18). Furthermore, apolipoprotein E (ApoE) on NP surfaces imitates LDL particles and uses LRP1-mediated transcytosis to enter the central nervous system. Confocal imaging and flow cytometry were used in a 2024 open-access study that used ApoE4-functionalized polystyrene NPs (80 nm) in a human iPSC-derived BBB model to show 2.1-fold increased transcytosis efficiency compared to ApoE3, with LRP1 knockdown lowering uptake by 67% (18) (Figure 2).

### 3.4 Evidence from human and animal studies

MP concentrations of 0.6–2.4 particles/g are found in human brain tissue according to autopsy investigations; dementia sufferers have 2–3 times greater loads than controls, and they are concentrated in the frontal cortex and hippocampus (15, 17, 29). After 90 days of exposure to 5 μm PS-MPs (0.1–10 mg/L in drinking water), rodent models show 0.03–0.12% brain translocation, with NPs building up in oligodendrocytes and causing α-synuclein oligomerization (identified by Western blot and Thioflavin-T staining), as well as myelin sheath disruption in the corpus callosum (18). Maternal NP exposure (50 nm PS-NPs, 0.5 mg/kg/day via oral gavage, gestational days 1–21) causes offspring brain accumulation (0.11% of maternal dose) at postnatal day 14, with cognitive deficits in Y-maze spontaneous alternation (≥38%) and anxiety-like behavior in the open field test (center time ≥51%). These effects are mediated by placental transfer (NPs found in milk-derived exosomes using Nanoparticle tracking analysis (NTA) and fluorescence labeling). These processes highlight how co-exposures and particle heterogeneity contribute to CNS translocation. Nevertheless, measuring exposure of MNPs to human brain remains challenging; standardized dosimetry, long-term cohort studies, and sophisticated imaging (such as fluorescence correlation spectroscopy) are required (16) (Table 1).

**Table 1:** Mechanisms of compromised gut permeability, gut-brain axis disruption, and MNPs-induced gut dysbiosis resulting in neurotoxicity.

Aspect	Experimental models	Key changes induced by MPs/NPs	Mechanisms/consequences	Relevant references
Composition of the gut microbiota (dysbiosis)	Mouse (C57BL/6), polystyrene microplastics (5 μm)	Beneficial taxa include butyrate-producing bacteria like *Faecalibacterium, Roseburia*, and *Bifidobacterium*. Pathogenic/opportunistic taxa include *Desulfovibrio spp*. and *Proteobacteria*.	Decreased microbial diversity; decreased synthesis of short-chain fatty acids (SCFA), particularly butyrate; increased production of hydrogen sulfide and lipopolysaccharide (LPS).	(3, 4, 23, 29)
Integrity of the intestinal barrier (“leaky gut”)	Mouse (C57BL/6), polystyrene microplastics (5 μm)	Increased intestinal permeability (3–5 × by FITC-dextran assay); 40%−70% downregulation of tight junction proteins (ZO-1, occludin, claudins).	Physical abrasion with dysbiosis-induced inflammation; adsorbed phthalates inhibiting PPAR-γ; thinning of the mucus layer.	(2, 4, 18)
Routes of translocation from the gut to the circulation/CNS	Mouse PS (5 μm)	1. Endocytosis (mostly NPs < 200 nm via M cells or enterocytes) 2. Leakage between cells (via weakened tight junctions) 3. Trojan horse (phagocytosis in GALT by monocytes, macrophages, and dendritic cells).	Immune cell transmigration across the blood-brain barrier; accelerated by oxidative stress and inflammation; size, charge, and surface chemistry-dependent uptake.	(15, 32)
Disruption of the gut-brain axis	Mouse model polystyrene nanoplastics (80–100 nm)	Changes in vagus nerve signaling; translocation of LPS and cytokines (such as TNF-α and IL-1β); decreased SCFA-mediated anti-inflammatory signaling; and changes in microbial metabolites (such as tryptophan and bile acids).	Increased BBB permeability, systemic inflammation, and neuroinflammation through NF-κB/NLRP3 pathways and microglial activation.	(4, 17, 18)
CNS/neurological results	Mouse (C57BL/6) Mouse model polystyrene nanoplastics (80–100 nm) Zebrafish (*Danio rerio*) Polystrene	MP/NP accumulation (2.4–7.5 × greater in dementia patients) in the hippocampus, frontal brain, and substantia nigra; oxidative stress (ROS, MDA); mitochondrial dysfunction; protein aggregation (Aβ, α-synuclein); and neurotransmitter imbalance (serotonin, dopamine).	Cognitive/motor impairments; transgenerational consequences; accelerated neurodegenerative-like disease (AD, PD, ALS-like).	(11, 15, 17, 32, 59)

## 4 CNS cellular and molecular toxicity

Microplastics (MPs) and nanoplastics (NPs) cause significant cellular and molecular toxicity in the brain's central nervous system (CNS) through oxidative stress, mitochondrial impairment, chronic neuroinflammation, synaptic dysfunction, neurotransmitter imbalance, and protein misfolding after they are translocated across the blood-brain barrier (BBB). Particle size (less than 100 nm NPs exhibit the maximum absorption), surface charge (positively charged particles increase toxicity two to five times), polymer type, and co-adsorbed environmental pollutants all worsen these pathways (2, 15–17, 33). MP/NP exposure is linked to accelerated neurodegenerative disease *in vitro, in vivo*, and emerging human data (Table 1).

In addition to their direct particle impacts, microplastics and nanoplastics (MNPs) act as reservoirs for leachable additives and degradation byproducts released during gastrointestinal (GI) transit, extended tissue retention, or following adhesion and aggregation in organs (18, 19). Under simulated GI circumstances, common chemicals like phthalates (like DEHP), bisphenol A (BPA), and flame retardants (like TBBPA) easily leach (15, 17, 36).

Through adsorption and prolonged retention, MPs improve additive bioavailability in the GI tract. In Caco-2 cells and human gut microbiota, co-exposure to polyethene (PE) MPs (5 μm, 10–100 μg/mL) with TBBPA (20 μM) increased oxidative stress and mitochondrial dysfunction by around 40% when compared to TBBPA alone (19, 37). Similarly, under GI simulations, phthalate-containing PVC MPs release 18%−34% of DEHP, which increases epithelial permeability and disrupts tight junction proteins (ZO-1, occludin, and claudins) (17).

**Figure 3:**
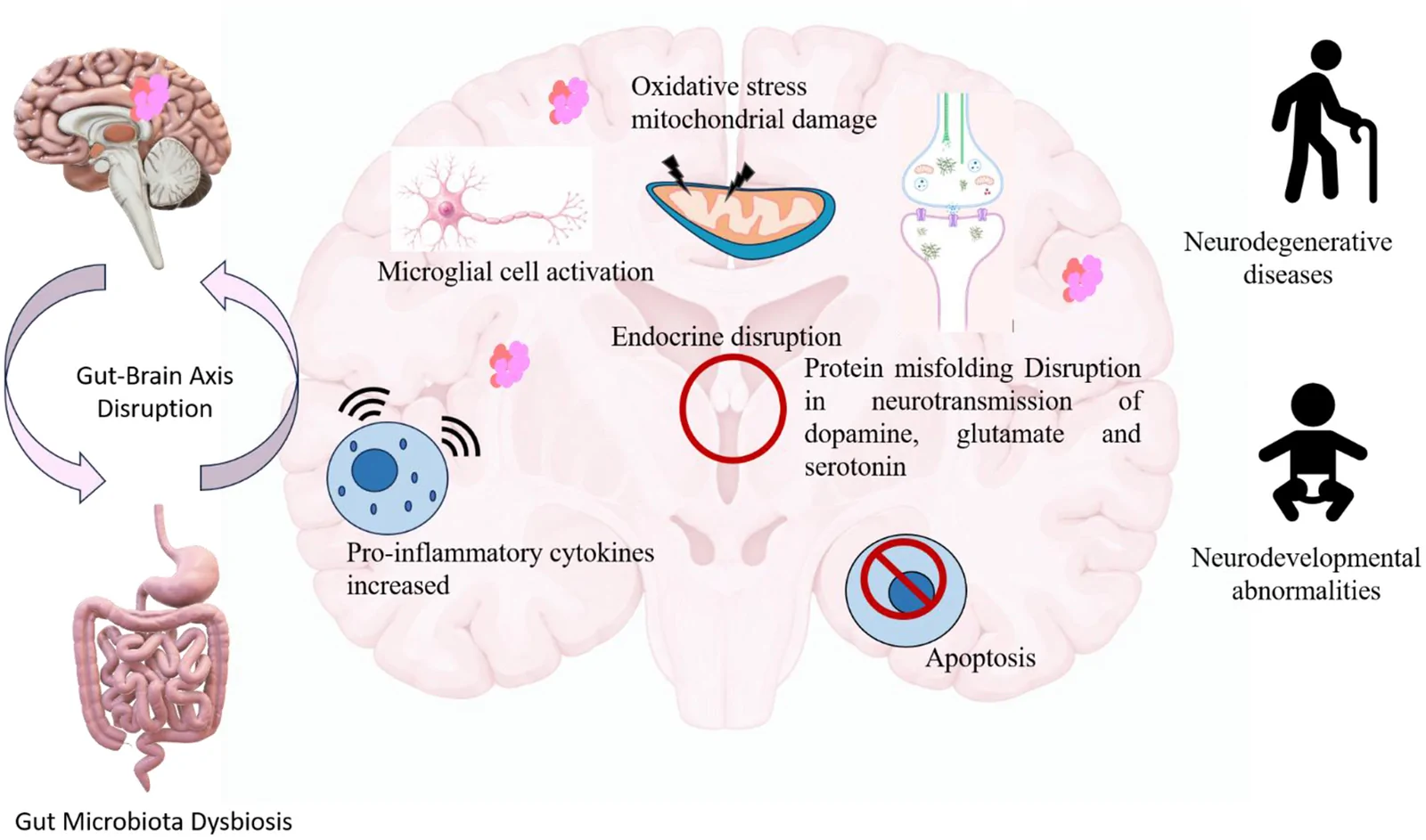
**Illustration showing accumulation of MNPs in brain tissues, Gut-brain axis disruption by MNPs and their neurotoxicity leading to activation of various pathological pathways to cause neurodegenerative diseases, Endocrine dysfunction and neurodevelopmental abnormalities**. MNPs enter the body and change the makeup of the gut microbiota. This damages the blood-brain barrier and causes oxidative stress (ROS), inflammatory responses, impaired autophagy, and inhibition of AChE in the brain, all of which lead to neurodegenerative alterations and anomalies in neurodevelopment. (The figure has been incorporated in the manuscript is generated with the help of Ai tool from figurelabs.ai).

Leached chemicals intensify neuroinflammation after they are either deposited in brain tissues through GBA signaling or translocated systemically across the weakened intestinal barrier. Phthalates and BPA exacerbate microglial activation, BBB permeability, and synaptic dysfunction by promoting excessive ROS production, NF-κB activation, and pro-inflammatory cytokine release (TNF-α, IL-1β, and IL-6) (18, 19, 38) (Figure 3). Postmortem investigations verify increased levels of BPA and phthalates in the Cerebrospinal fluid (CSF) of AD and PD patients, which are associated with 2.4–7.5 times greater MP/NP loads in dementia cases and co-localization with aggregates of amyloid-β and α-synuclein (11, 15).

Compared to clean particles, MP-contaminant complexes cause more dopaminergic neuron death, α-synuclein oligomerization, mitochondrial dysfunction, and NLRP3/NF-κB-mediated neuroinflammation in preclinical models (16, 17). By increasing tissue adhesion and additive release, surface charge and age further regulate leaching (18, 19) (Table 1). Thus, MNPs not only affect the brain tissues rather it carries various other pollutants that collectively cause neuroinflammation.

### 4.1 ROS generation and oxidative stress

Reactive oxygen species (ROS) in brain cells are quickly increased by MP/NP treatment, upsetting the redox equilibrium. BV-2 microglial cells treated with 50 nm polystyrene nanoparticles (PS-NPs) at 20 μg/mL for 24 h showed a 2.8-fold increase in ROS (DCFH-DA test), 45% Reduced glutathione (GSH) depletion, and 32% Superoxide dismutase (SOD) inhibition (16). When treated with 1 μm PS-MPs (10 μg/mL, 48 h), SH-SY5Y human neuroblastoma cells exhibit a 3.1-fold increase in Malondialdehyde (MDA) and a 2.4-fold increase in 8-OHdG, suggesting lipid and DNA oxidation (8, 9). Due to increased cellular absorption, positively charged amine-modified NPs produce two to five times as much ROS as their carboxylated counterparts (16, 33). Co-exposure to adsorbed lead (Pb) increases the production of hydroxyl radicals. 4.2-fold in primary rat neurons using Fenton reactions (18) (Figure 3).

### 4.2 Mitochondrial dysfunction

When NPs colocalize with mitochondria, they interfere with Adenosine triphosphate (ATP) production, membrane potential, and electron transport. When zebrafish larvae are exposed to 60 nm fluorescent PS-NPs (0.5 mg/L, 72 h), they exhibit mitochondrial accumulation in hippocampus neurons, a 38% decrease in ΔΨm (JC-1), and a 51% decrease in ATP. Transmission electron microscopy (TEM) shows that N2a cells treated with 100 nm PEGylated NPs (5 μg/mL) had swollen mitochondria and lost cristae. Polymerase γ inhibition causes a 29% reduction in cortical mtDNA copy number in rodents exposed to 5 μm PS-MPs (0.1 mg/L, 90 days) (4) (Figure 3).

### 4.3 Neuroinflammation and microglial activation

Through the NLRP3 inflammasome and NF-κB pathways, MNPs activate astrocytes and microglia. When primary rat microglia are exposed to 0.5–5 μm PS-MPs (20 μg/mL, 24 h), they increase NO (2.9-fold), TNF-α (3.8-fold), and IL-1β (4.5-fold) (16). The child hippocampus had 42% more Iba1+ microglial nodules after maternal NP exposure (50 nm, 0.5 mg/kg/day) (2). Astrocytes generated from human iPSCs exhibit GFAP. 2.1-fold and CXCL10 about 3.3-fold when using ApoE4-NPs (Figure 3). On exposure of MNPs to brain tissues, Oxidative stress, mitochondrial failure, and neuroinflammation occur as interconnected events. As ROS induces mitochondrial dysfunction, it later leads to chronic inflammation.

mNPs accumulation in the brain causes interconnected events such as oxidative stress, mitochondrial failure, and neuroinflammation, which leads to neurodegenerative disorders.

### 4.4 Particular microbial taxa in MNP-induced dysbiosis and their connections to neurodegeneration

Exposure to MNP causes gut dysbiosis, which is characterized by an increase in opportunistic Gram-negative bacteria and a decrease in beneficial *Firmicutes* taxa. This promotes intestinal barrier breach, systemic inflammation, and disturbance of the gut-brain axis, which speeds up neuroinflammation and dementia (4, 39).

*Faecalibacterium prausnitzii* and *Roseburia spp*. (butyrate-producing Firmicutes) and *Akkermansia muciniphila* (mucin degrader) are important deficient taxa. Short-chain fatty acid (SCFA) synthesis is reduced, tight junction proteins (ZO-1, occludin) are compromised, and the mucus layer is compromised, allowing MNP and LPS translocation (6). This is further worse by positively charged particles and small PS-NPs ( ≤ 100 nm), which selectively damage ROS-sensitive Firmicutes while preferring robust Bacteroidetes (6, 20, 39).

NF-κB/TLR4-mediated inflammation and vagus nerve signaling are amplified when Desulfovibrio spp. and Proteobacteria overgrow, increasing hydrogen sulfide and LPS (40, 41). In important brain areas, this dysbiotic profile increases MNP paracellular and endocytic absorption, systemic circulation, and BBB crossing, leading to microglial activation, increased cytokines (TNF-α, IL-1β), oxidative stress, and aggregation of pathogenic proteins (α-synuclein, Aβ) (7, 42, 43) (Figure 2).

These changes at the taxonomic level are similar to those seen in models of Parkinson's and Alzheimer's disease, where increased LPS and decreased SCFA signaling cause neuroinflammation and neuronal death (4, 44). Polymer- and dose-dependent dangers are highlighted by the milder but cumulative impacts of low-dose, environmentally relevant exposures (39).

## 5 Preclinical and clinical evidence linking MNPs to neurodegeneration

Microplastics (MPs) and particularly nanoplastics (NPs) are strongly linked to neurotoxic outcomes that resemble the pathophysiological characteristics of major neurodegenerative diseases, such as Alzheimer's disease (AD), Parkinson's disease (PD), amyotrophic lateral sclerosis (ALS), and dementia in general, according to an increasing amount of preclinical (*in vitro* and *in vivo*) and emerging clinical evidence. Chronic neuroinflammation, oxidative stress, mitochondrial dysfunction, protein aggregation, synapse loss, and disturbance of the gut–brain axis are the main mechanisms mediating these consequences.

### 5.1 Rodent models: Parkinson's-like and Alzheimer's-like pathology

Mice and rats exposed to polystyrene micro- and nanoplastics orally over an extended period of time consistently develop brain buildup and neurodegenerative hallmarks. Long-term exposure to 1–5 μm MPs or 50–100 nm NPs (0.01–10 mg/L in water intake for 90–180 days) causes identifiable particle translocation into the brain, α-synuclein oligomerization, substantial loss of dopaminergic neurons in the substantia nigra, decreased striatal dopamine levels, and obvious motor deficits on rotarod and open-field tests, which closely resembles the pathology of Parkinson's disease. Key features of Alzheimer's disease are mirrored in the Morris water maze by the same exposure paradigms, which cause amyloid-β (Aβ1–42) deposition, tau hyperphosphorylation at AD-relevant sites (AT8, PHF-1), hippocampus neuronal death, and significant spatial memory impairment. Extensive myelin sheath breakdown, oligodendrocyte death, and white-matter rarefaction in the corpus callosum are additional symptoms of prolonged exposure (6 months) that are similar to those of multiple sclerosis and amyotrophic lateral sclerosis (45).

### 5.2 Mammalian developmental and generational consequences

When a mother is exposed to nanoplastics during pregnancy and breastfeeding, NPs are transferred across the placenta and accumulate in the brains of the fetus and newborn. Offspring of dams orally gavaged with 50 nm polystyrene NPs (0.5–5 mg/kg/day, gestational days 1–21) show reduced hippocampal neurogenesis, anxiety-like behavior, elevated pro-inflammatory cytokines, microglial activation (42%−60% increase in Iba1^+^ nodules), persistent hippocampal and cortical NP burdens, and long-lasting cognitive deficits that persist into adulthood (29). These results raise concerns about the acceleration of neurodegenerative risk across generations and identify a crucial window of susceptibility.

### 5.3 Zebrafish models: quick translocation and real-time neurotoxicity imaging

When exposed to fluorescent 25–100 nm polystyrene NPs (0.1–5 mg/L), zebrafish larvae and adults exhibit rapid blood–brain barrier crossing within hours, specifically buildup in dopaminergic regions, hypo-locomotion, loss of tyrosine hydroxylase-positive neurons (28%−40%), α-synuclein aggregation, fragmentation of the mitochondria via Drp1 upregulation, and increased expression of amyloid-β42 and hyperphosphorylated tau (11, 46, 47). At ecologically relevant doses, our models offer direct visual evidence of particle translocation and early-stage Parkinsonian and Alzheimer-like molecular disease.

Evidence from rodent and zebrafish models demonstrates that MNPs accumulation in the brain mimics the major molecular and cellular processes of neurodegenerative diseases. This process occurs by inducing oxidative stress, mitochondrial malfunction, chronic neuroinflammation, protein aggregation, and GBA disruption. Taken together, the experimental findings support that MNPs are environmental contaminants that contribute to neurodegenerative disease. Still, human causal evidence and the impact of long-term exposure are yet to be revealed.

### 5.4 Brain organoids and models produced from human iPSCS

Models developed from human induced pluripotent stem cells (hiPSCs) provide essential translational platforms for assessing the neurotoxicity of micro- and nanoplastics (MNPs). Polystyrene microplastics (PS-MPs, 1–10 μm; 5–100 μg/mL) have biphasic, time- and size-dependent effects in hiPSC-derived cortical spheroids: short-term exposure (days 4–10) increases neural progenitor proliferation with upregulation of Ki67, Nestin, and PAX6 along with oxidative stress responses, while prolonged exposure (up to 30 days) hinders neuronal differentiation (TUBB3, TBR1 downregulation), lowers cell viability, and alters organoid architecture (48).

Exposure to polystyrene nanoplastics (PS-NPs, < 100 nm; 50–200 ng/mL) or polypropylene nanoplastics (PP-NPs) causes mitochondrial dysfunction (structural changes, decreased membrane potential, and ATP production) in hiPSC-derived cerebral organoids. It also increases apoptosis, suppresses neuronal maturation (TUJ1, MAP2, and PAX6 downregulation), reduces organoid size, and impairs neural network activity (44, 45). Wnt signaling, mitochondrial homeostasis, and progenitor maintenance are all impacted by these abnormalities.

These findings are supported by complementary two-dimensional human cell line models. PS-NPs cause oxidative stress, mitochondrial impairment, decreased neurite outgrowth, and increased amyloid-β release in SH-SY5Y neuroblastoma cells in a manner dependent on surface charge and size (16, 62). Human neural stem cells (NSCs) exhibit effective endocytosis that results in cytoplasmic accumulation and death, whereas human cerebral microvascular endothelial cells (hCMEC/D3) exhibit MNP-induced barrier breakdown and increased permeability.

### 5.5 New human postmortem and clinical data

Micro- and nanoplastics have been found in 100% of investigated human brains (*n* > 50) in recent autopsy investigations (2024–2025), with concentrations of 0.6–2.4 particles/g wet weight in gray and white matter. Crucially, compared to age-matched neurologically healthy controls, those with clinically confirmed dementia (Alzheimer's, vascular, Lewy-body, or mixed) have MP/NP loads that are 2.4–7.5 times greater, with the hippocampus, frontal cortex, and substantia nigra showing the largest accumulation (15, 17, 29). Particles often co-localize with α-synuclein aggregates, neurofibrillary tangles, and amyloid-β plaques (45). Patients with AD and PD had higher levels of plastic-derived monomers (BPA, phthalates) and polymer fragments in their cerebrospinal fluid, according to small clinical cohorts. Higher projected lifetime MP consumption is associated with poorer MMSE scores and greater plasma neurofilament light chain, a sensitive biomarker of continuing neurodegeneration, according to a pilot longitudinal study of 480 aged people.

### 5.6 Synthesis and consequences

The basic pathogenic cascade of neurodegenerative diseases-oxidative stress, mitochondrial dysfunction, chronic neuroinflammation, protein misfolding, synapse loss, and neuronal death-is consistently induced by MPs and particularly NPs across species, exposure levels, and experimental systems. Chronic low-dose MP/NP exposure is a unique, modifiable environmental risk factor for accelerated neurological illness in aging populations, according to the convergence of rodent, zebrafish, human organoid, and now postmortem human findings.

As plastic pollution rise globally, the impact of MNPs on human health must be analyzed. The convergence of experimental models and human evidence suggests that MNPs are not mere pollutants in the environment but also as potential neurotoxicants that are affecting human health and longevity.

## 6 Mitigation strategies and policy implications

Targeted mitigation emphasizing source reduction, better waste management, and exposure minimization is supported by evidence connecting MNPs to disturbance of the gut–brain axis. Strategies are grouped according to the strength of the evidence and the state of execution.

The effectiveness of established regulations has been shown. Since 2015, some nations have imposed bans on microbeads in rinse-off cosmetics, which have greatly decreased environmental concentrations (22, 27). Intentionally introduced synthetic polymer microparticles are prohibited under the EU REACH Regulation (EU) 2023/2055, which will be phased in between 2025 and 2035 (22).

Major unintended sources, including textile fibers and tire wear particles, are the focus of proposed treatments. To lessen primary MP releases, extended producer responsibility (EPR) programs and design-for-circularity packaging concepts are being actively developed (22, 27).

Although real-world performance varies depending on particle properties and fouling, advanced wastewater treatment systems employing membrane bioreactors (MBR) and ultrafiltration achieve 88%−99.9% MP removal and 80%−97% NP removal in controlled and pilot experiments (49).

Following the INC-5 process in 2025, international discussions for a legally binding UN Plastics Treaty continue with the goal of supporting trash infrastructure in poor nations and achieving complete life-cycle management (27). Enforceable models are provided by regional frameworks like the EU Single-Use Plastics Directive.

Microfiber laundry filters, natural-fiber fabrics, and choosing glass or tap water over bottled sources are examples of individual-level strategies that are feasible in reducing personal exposure (28, 30).

Together with standardized monitoring procedures and the creation of health-based exposure limits, these evidence-based tactics offer a workable framework for lowering MNP burdens while filling up any remaining knowledge gaps.

### 6.1 Preventative and therapeutic approaches

To reduce MNP-induced gut dysbiosis, barrier disruption, and gut-brain axis neurotoxicity, targeted preventative and therapeutic approaches are essential. While directing cutting-edge mechanistic and clinical research, these approaches concentrate on reestablishing microbial balance, lowering oxidative stress, and maintaining barrier integrity. In MNP-exposed mice, probiotics (like Lactobacillus strains) and prebiotics (such galacto-oligosaccharides, or GOS) successfully restore beneficial taxa, improve tight junction proteins, decrease LPS translocation, and lessen neuroinflammation and behavioral impairments (50).

By restoring mitochondrial function, preventing excessive mitophagy, and reducing oxidative stress and motor deficits brought on by polystyrene nanoplastics, melatonin exhibits considerable neuroprotective potential (9).

These methods are useful for planning future research, such as long-term human trials that combine probiotics and prebiotics with MNPs biomonitoring, neuroimaging, and cognitive evaluations, as well as hiPSC-organoid co-exposure models to validate tailored interventions for populations at risk.

## 7 Knowledge gaps and future research directions

Despite the fast growth of data connecting micro- and nanoplastics (MNPs) to disturbance of the gut-brain axis and neurodegenerative processes, there are still significant gaps in our understanding that make it impossible to draw firm conclusions about human risk and to develop effective preventative measures. While real-world MNPs are extremely heterogeneous in terms of polymer composition (PE, PP, PET, PVC, etc.), shape (fragments, fibers, films), degree of weathering, and surface-added chemical pollutants (heavy metals, PAHs, phthalates, BPA), the majority of mechanistic studies still rely on pure, spherical polystyrene particles of uniform size and charge (16, 18, 22, 26). This artificial homogeneity significantly underestimates neurotoxicity, BBB penetration, and real-world translocation efficiency (35, 51). To increase translational relevance, environmentally aged, multi-polymer combinations with realistic co-exposures should be given priority in future toxicological research.

The lack of genuine chronic, low-dose, lifetime exposure trials is a second significant drawback. Acute or sub-chronic exposures at doses orders of magnitude greater than expected human consumption (0.1–5 g/week) provide almost all of the preclinical neurotoxicity evidence currently available (21, 28, 35). Extended (≥1–2 years) To capture cumulative effects, age-related vulnerability, and progressive neurodegenerative disease, rodent studies and lifetime models in shorter-lived species utilizing environmentally appropriate dosages are crucial (2, 52).

The underrepresentation of vulnerable groups persists. The most vulnerable groups are probably those with pre-existing gut dysbiosis or weakened BBB, the older adult(s), APOE4 carriers, and exposure during pregnancy and early life, although there are hardly any specific cohorts (29, 53). To determine key windows of vulnerability and measure dose-response interactions, large prospective birth cohorts and geriatric cohorts incorporating serial biomonitoring, neuroimaging, and cognitive evaluation are desperately needed (53, 54).

Another crucial obstacle is the standardization of analytical techniques and human biomonitoring procedures. Exposure assessment and inter-study comparability are significantly hampered by the wildly variable results produced by current detection methods (55, 56). To enable comparable exposure data and science-based regulatory limits, the creation of globally standardized, open-source analytical protocols that accurately measure particle number, size distribution, polymer type, shape, and bound chemical additives in food, drinking water, air, and human matrices (blood, brain tissue, placenta, feces, hair) has become a top priority (57, 58).

Lastly, MNPs concentrations in the brains of people with dementia are typically three to ten times greater than those of age-matched neurologically healthy controls, according to recent postmortem investigations (15, 17, 59). These cross-sectional findings are insufficient to establish causality. To establish dose-response relationships, confirm causality, and quantify the true contribution of MNPs to the increasing global burden of neurodegenerative disease, large-scale, prospective epidemiological cohorts that combine repeated biomonitoring of plastic fragments and additives, superior neuroimaging (amyloid/tau PET, neurofilament light chain), genetic risk profiling (e.g., APOE genotype), and detailed reconstruction of entire-life plastic exposure (53, 60).

### 7.1 Omics approaches for clarifying molecular mechanisms and toxicokinetic elements of MNPs on the gut-brain axis

To understand the intricate toxicokinetics and mechanisms by which micro- and nanoplastics (MNPs) interfere with the gut-brain axis (GBA), multi-omics approaches are crucial from a molecular and microbiological standpoint. Comprehensive profiling of gut microbial communities is made possible by metagenomics, which reveals MNP-induced dysbiosis marked by enrichment of opportunistic pathogens like *Desulfovibrio spp*. and *Proteobacteria* and decreased abundance of beneficial short-chain fatty acid (SCFA)-producing taxa like *Faecalibacterium, Roseburia*, and *Bifidobacterium*. These changes promote intestinal barrier dysfunction and systemic translocation of MNPs and microbial metabolites by altering functional gene pathways involved in SCFA biosynthesis, lipopolysaccharide (LPS) generation, and xenobiotic degradation (6, 61).

Key metabolite disruptions that connect gut dysbiosis to neurotoxicity are identified using metabolomics, including targeted and untargeted LC-MS techniques. Polystyrene nanoplastic exposure disrupts bile acid, nucleotide, carnitine, and energy balance pathways in both intestinal and brain tissues, depletes SCFAs (such as butyrate), increases pro-inflammatory mediators, and changes tryptophan metabolism toward the kynurenine pathway. These alterations are correlated with neuroinflammatory lipid profiles and neurotransmitter abnormalities (such as decreased serotonin) (50, 61).

Particle-specific toxicokinetics and host responses are further revealed by transcriptomics and proteomics. In enterocytes and blood-brain barrier endothelial cells, MNPs upregulate genes and proteins linked to endocytosis and transcytosis (such as clathrin, caveolae, and LDLR/LRP1 pathways) while downregulating tight junction elements (ZO-1, occludin). They trigger NF-κB/NLRP3 inflammasome signaling, protein aggregation cascades (Aβ, α-synuclein), oxidative stress (ROS/MDA pathways), and mitochondrial dysfunction (e.g., altered mtDNA copy number, AMPK/ULK1-mediated mitophagy) in brain tissues (9, 14).

## 8 Conclusion

Microplastics (MPs), and particularly nanoplastics (NPs), are ubiquitous and pernicious environmental concerns with significant consequences for brain health, according to convergent data from environmental, toxicological, preclinical, and developing clinical investigations. Every environmental compartment and human physiological system is contaminated by these particles, which are produced at an industrial scale surpassing 400 million tons of plastic yearly and are expected to accelerate under existing trajectories. Current preclinical evidence consistently demonstrates that exposure of MNPs through oral routes accelerates the gut dysbiosis process, diminishes short-chain fatty acids (SCFAs), induces oxidative stress, compromises epithelial barrier integrity, and activates inflammatory pathways, including NF-κB and NLRP3 inflammasome signaling. Alteration in the intestinal environment results in systemic effects via increased intestinal permeability, translocation of microbial products like lipopolysaccharides, release of cytokines, metabolites and also causes vagal dysfunction. Micro and Nanoparticles also directly penetrate through the blood-brain barrier and accumulate in the brain tissues. Together, these factors lead to neuroinflammation, synaptic impairment, mitochondrial dysfunction, and cognitive and behavioral abnormalities.

The results of recent human postmortem analyses (2024–2025) that show universal brain contamination are especially concerning. MNP burdens are 2.4–7.5 times higher in dementia patients than in age-matched controls, and they frequently co-localize with amyloid-β plaques, neurofibrillary tangles, and α-synucleic aggregates. Chronic low-dose MNP exposure is positioned as a novel, modifiable accelerator of the global neurodegenerative epidemic due to transgenerational transfer via placental and lactational routes, increased vulnerability during neurodevelopmental windows, and in aging or genetically susceptible populations (e.g., APOE4 carriers). Despite increasing evidence on the role of MNPs in gut-brain disruption, a robust causal link to degenerative disease in humans due to exposure is yet to be established. Current knowledge is mostly derived from experimental models, which suggest a single mechanistic pathway or the factors. No such studies are exploring the cascade from intestinal exposure to neurological impairment. Furthermore, existing studies primarily design the investigation by using pristine polystyrene particles with short-term exposure and at higher concentrations that found in the environment. Therefore, these experimental models do not adequately mimic the real-time exposure of MNPs by humans. Due to a lack of standardized analytical methods, the disease causation on exposure to MNPs is not deciphered.

Addressing these limitations can lead to future research being conducted effectively. Establishment of standardized analytical methods to detect MNPs, particularly NPs, preparation of experimental models that are suitable for chronic low-dose exposure as occurs in real-time and integrated multiorgan experiments, and an integrated multi-organ experimental system to explore the gut dysbiosis to neurological impairment simultaneously. Microbial profiling, a multiomics approach in human cohort are important for establishing the causal relationship, deciphering molecular cascades and establishing the biomarkers for MNPs exposure.

Coordinated source-reduction strategies that have previously proven effective (microbead bans, microfiber-filter mandates), improved waste-management infrastructure, sophisticated wastewater treatment, and implementation of the 2025 UN Plastics Treaty clauses can all lead to effective mitigation. Individuals may immediately reduce their exposure by reducing the amount of plastic they come into contact with in food, water, clothes, and interior surroundings. To measure attributable risk and set evidence-based criteria, it is still crucial to prioritize large prospective human cohorts that include serial MP/NP biomonitoring, neuroimaging, genetic profiling, and cognitive evaluation.
